# U. S. V. M. A.

**Published:** 1897-10

**Authors:** 


### SOCIETY PROCEEDINGS.

### U. S. V. M. A.

### THE STORY OF NASHVILLE.

*The Journey.*—For some five days prior to the opening of the Thirty-fourth Annual Convention there reached Nashville from the remotest sections of our country the most representative body of veterinarians that has ever gathered together on American soil, all imbued with great determination to give their best efforts for the consideration of those subjects of so much importance to the welfare of the profession. Before convention days had arrived the State of Washington was vieing with Massachusetts in their interest in coming events. Pennsylvania, from its most eastern and western points, had its devotees there to friendly contest with the Empire State their relative strength in numbers in attendance. Louisiana was early on the field, and, as she referred to the others yet to come, she smiled as she noted on every side the keen interest of the South in this first gathering in its territory. The Central West sent her most representative members of the profession, and with those from more northern points there was such a rivalry as to distances travelled that one would want to know whether there could be additional miles to the journeys so many had made to this Mecca for 1897. Down from the "Hub," from the "Metropolis," from the "City of Churches," the "Quaker City," the "Monumental City of the South," and the "City of Magnificent Distances" there rolled over the tracks of the Southern Railway, through the beautiful "Land of the Sky," so well and appropriately named, many of those whose faces are ever looked for and always welcomed at our annual gatherings; from the "Windy City," "Forest City," "Queen City of the West," "Railroad City," "Falls City," and "Mound City," down the Mississippi Valley, every train from distant points added to the gathering crowd which has made Nashville famous in Association circles. The doors of hospitality of this beautiful city, nestling within the confines of hilly and mountainous country on all sides, swung inward for the veterinary profession until their creaking hinges would fain have broken if there was anything additional needed to insure our welcome, make pleasant our stay, and to speed our parting with such a wealth of pleasant, happy recollections of the Southern hospitality enjoyed in the "Rock City."

*Who Were There.*—Drs. E. B. Ackerman, Brooklyn, N. Y.; A. H. Baker, Chicago, Ill.; R. R. Bell, Brooklyn, N. Y.; R. H. Bird, Helena, Mont.; T. S. Butler, Starkville, Miss.; C. A. Cary, Auburn, Ala.; A. W. Clement, Baltimore, Md.; J. W. Connoway, Columbia, Mo.; T. B. Cotton, Mt. Vernon, Ohio; W. H. Dalrymple, Baton Rouge, La.; Charles Ellis, St. Louis, Mo.; H. D. Gill, New York City; J. O. Greeson, Kokomo, Ind.; T. D. Hinebauch, Fargo, N. D.; N. P. Hinkley, Buffalo, N. Y.; W. Horace Hoskins, Philadelphia, Pa.; W. H. Kelly, Albany, N. Y.; James Law, Ithaca, N. Y.; W. H. Lowe, Paterson, N. J.; M. H. McKillip, Chicago, Ill.; J. C. Meyer, Jr., Cincinnati, Ohio; S. B. Nelson, Pullman, Wash.;

F. H. Osgood, Boston, Mass.; E. P. Niles, Blacksburg, Va.; W. B. Niles, Ames, Iowa; J. M. Parker, Haverhill, Mass.; Leonard Pearson, Philadelphia, Pa.; T. B. Pote, Terre Haute, Ind.; W. C. Rayen, Nashville, Tenn.; James B. Rayner, West Chester, Pa.; T. B. Rayner, Philadelphia, Pa.; D. E. Salmon, Washington, D. C.; J. W. Schiebler, Memphis, Tenn.; F. S. Schoenleber, Morris, Ill.; E. H. Shepard, Cleveland, Ohio; M. Stalker, Ames, Iowa; S. Stewart, Kansas City, Kan.; A. S. Wheeler, Biltmore, N. C.; T. E. White, Sedalia, Mo.; W. L. Williams, Ithaca, N. Y.; J. F. Winchester, Lawrence, Mass.; A. W. Bitting, Lafayette, Ind.; George B. Blackman, Rome, Ga.; S. H. Caldwell, Chicago, Ill.; R. H. Drummond, Birmingham, Ala.; T. Eisenman, Louisville, Ky.; H. D. Fenimore, Knoxville, Tenn.; M. Francis, College Station, Texas; C. A. Geddes, Tenn.; J. M. Good, Chattanooga, Tenn.; C. W. Heitzman, Jr., New Orleans, La.; J. W. Jamieson, Paris, Ky.; J. R. Mitchell, Evansville, Ind.; M. O'Connell, Holyoke, Mass.; Joseph Plaskett, Nashville, Tenn.; E. M. Ranck, Philadelphia, Pa.; J. C. Robert, Agricultural College, Miss.; J. C. Foelker, Allentown, Pa.; N. Rectenwald, Pittsburg, Pa.; J. C. Blankton, Farmville, Tenn.; G. D. Bray, Columbia, Tenn.; R. E. Collins, Memphis, Tenn.; T. H. Hagyard, Nashville, Tenn.; S. E. Jago, Shelbyville, Tenn.; J. P. Rauch, Memphis, Tenn.; T. W. Scott, Nashville, Tenn.; R. J. Michener, Lebanon, Ohio; F. S. Roop, Blacksburg, Va.; C. Barnwell Robinson, Washington, D. C.; D. K. Smith, Toronto, Ontario; George R. White, Chapel Hill, Tenn.; F. Abele, Jr., Quincy, Mass.; J. E. Aldrich, Memphis, Tenn.; G. E. Browning, Boston, Mass.; B. F. Cockrill, M. D., Nashville, Tenn.; L. E. Morgan, M.D., Baton Rouge, La.; J. D. Plunkett, M.D., Nashville, Tenn.; Ferdinand Smith, Chicago, Ill.; Mr. Harold Sorby, of the Pasteur Vaccine Co., Chicago, Ill.; Mr. Frederick Krey, representing the firm of John Reynders & Co., New York City; N. P. Hinkley and son, Buffalo.

The ladies present were Mrs. A. H. Baker, R. R. Bell, J. W. Connoway, T. Bent Cotton, M. Francis, H. D. Fenimore, H. D. Gill, T. H. Hagyard, John R. Hart, W. Horace Hoskins, M. H. McKillip, F. H. Osgood, Rapin, W. C. Rayen, C. B. Robinson, E. H. Shepard, F. S. Schoenleber, Misses Breast, Hogan, Miller, Mills, McDonnald, Rectenwald, Watson.

*What was Done at Nashville.*—The welcome of the press at Nashville was very cordial, indeed, and the exhaustive daily reports of the *American* and *Banner* were very much appreciated by all who were present. The *Banner* daily reproduced on its pages photographs of many of the members, among others Drs. Osgood, Cary, Stewart, Williams, Hoskins, R. R. Bell, T. B. Rayner, James B. Rayner, Foelker, Ackerman, Gill, Hinkley, Stalker, and Pearson. That ever-to-be-remembered kindly and public-spirited chairman of the Entertainment Committee of the Exposition, Major John J. McCann, whose cheerful face and sunny disposition charmed all with whom he came in contact, was ever and anon present to add to the wealth of pleasure and enjoyment of our stay in Nashville. His attention to the ladies while our sessions continued, their conveyance to the Exposition in special cars, when the gates were lifted high for their entrance, and every door thrown open for their admission, not to speak of the feast spread so bounteously that they might satisfy every desire, are memories that leave impressions never to be forgotten and which make a country one in the common impulses, hopes, and aims that bind its people together.

The city's chief executive was represented on the morning of the opening of the convention by the Rev. W. T. Haggard. His welcome on behalf of the city was very cordial, and in that Nashville was the first Southern city to receive the honor of our convening that it was fully appreciated. As representative of Governor Taylor and the Centennial Exposition, Major John J. McCann extended the members and delegates the most hearty welcome, and with the warm heart of a true Southern entertainer he won by his greeting the close attention and the thorough appreciation of all that they were in the house of their friends. On behalf of the Association, Dr. Leonard Pearson responded, referring to our high expectations and hopes, and how rapidly and completely they were being realized. He referred to the high place Tennessee held in the ranks of breeders of valuable and high-bred live-stock, and that it was well for us to assemble at such a centre. Touching upon the importance of the live-stock industry, he said that if all domestic animals were destroyed great suffering would ensue. "There would be no horses or mules to till the fields; no beef, mutton, pork, milk, or eggs; no wool; no means of transportation in many sections, and no manure with which to maintain the fertility of the soil, under which conditions all business would stagnate and perish, the people suffer for want of food, and the land would become sterile. These were potent reasons why veterinary science is of the highest importance to the South, and the need of more and the best men of our profession in public positions was a question of vital importance." Speaking for the many representative veterinarians of the South for a generous recognition and support, he thanked the State, the city, and the Exposition for the warm welcome, and was already assured of a large and profitable meeting.

President Osgood then, in one of the most polished and scholarly addresses ever presented to the Association, gave thorough evidence of how well he had studied and considered the welfare of the Association, and made many salient suggestions for better enlisting the active interest of the entire membership—calling attention to the fact that a new President had to wait one year before he could outline his policy; suggesting for consideration plans to enable members to vote by proxy; to assist in the suggestion of suitable candidates for the elective officers; recommending greater uniformity in State laws and mutual recognition of certificates of State Boards of Examiners, and many other equally vital recommendations for the welfare and advancement of the interests of this organization as the national representative of the profession.

The Executive Committee was early to work on September 6th, with Drs. Osgood, Stewart, Hoskins, Hinkley, Williams, Cary, Bell, Pearson, and Stalker present, and Drs. Howard, Robertson, and Trumbower absent; as temporary appointees, Drs. Rayen and Gill. Chairman Hoskins called the meeting to order. The favorable recommendation for membership of the following-named applicants added this new strength to Association members: Ira K. Atherton, Marshalltown, Iowa; George B. Blackman, Rome, Ga.; S. H. Caldwell, Montgomery, Ala.; P. J. Cronon, Boston, Mass.; R. H. Drummond, Birmingham, Ala.; M. Francis, College Station, Texas; Joseph M. Good, Chattanooga, Tenn.; C. W. Heitzman, Jr., New Orleans, La.; D. King, Natchez, Miss.; C. N. Kinnell, Pittsfield, Mass.; J. C. Norton, Phœnix, Ariz.; A. W. Bitting, Lafayette, Ind.; Joseph Plaskett, Nashville, Tenn.; E. M. Ranck, Philadelphia; J. C. Robert, Agricultural

College, Miss.; G. R. White, Chapel Hill, Tenn.; D. P. Frame, Colorado Springs, Colo.

The resignations of Drs. Herbert Neher and Henry F. Leonard were accepted by the convention, and they will no longer mingle their names on the roll of honor of the national organization. The granting of the resignation of Prof. Theobald Smith was followed by his recommendation and unanimous election to honorary membership.

The charges of violation of the Code of Ethics in unprofessional advertising were not sustained against Dr. John Faust, of Poughkeepsie, who retains his name among the list of faithful ones whose many years have ever sustained every responsibility placed upon his shoulders.

The utmost spirit of leniency prevailed among all toward the delinquents, and with a few exceptions additional time was recommended and given. Where desired, their names were temporarily suspended, to avoid accumulations of additional dues, with power to reinstate by the Secretary on payment of dues already accrued.

The request of Dr. James B. Rayner for an investigation of certain published statements detrimental to his character in the *American Veterinary Review* for August, 1897, over the signature of Dr. James Law, charging him, on alleged early statements of that periodical, with connection with the sale of diplomas, was granted and a committee appointed, consisting of Drs. Bell, Williams, and Pearson, with the instructions that they report at the next meeting of the Association, or earlier, through the veterinary periodicals, if their investigation warrants the same.

The Executive Committee have cited Dr. Gerald Griffin, of Fort Reno, Indian Territory, to appear before them and answer charges of violation of the Code of Ethics in advertising secret formulas of remedies; also Dr. J. H. Wattles, of Kansas City, Mo., for violation of the code.

Drs. Hempleman, McClellan, G. T. Netherton, and M. A. Piche sent in their resignations; but as they are in arrears with their dues, the Secretary was instructed to inform them that their resignations cannot be acepted until these arrearages are liquidated.

The reports of the various standing committees followed, showing the completion of the local arrangements. The finances of the Association were shown to be in better condition than for several years, exhibiting a surplus over all liabilities of $852.44. This healthy condition of Association finances called from the Secretary in his report a recommendation that we consider the advisability of reducing the dues from five to three dollars.

The Publication Committee in its report reviewed the cost of publishing the proceedings in the past, and, in estimating the cost for the present meeting, said that at least eighty-five dollars would be saved compared with the cost of 1896, based upon the same number of pages and character of work. A saving was also promised in the stenographic report of the meeting, and the proceedings complete, ready for distribution, in thirty days after delivery of matter to the printer; and as this latter was accomplished before September 15th, the members will receive their full reports by October 15th.

The Committee on Diseases, through the chairman, Dr. Theobald Smith, based their report on what seemed to be the field for this body, and urged special work only or the wiping away of this committee. The discussion and consideration of the report was to retain this committee, and that it should during the coming year make special efforts to gain information as to the extent, prevalence, and characteristics of outbreaks of osteoporosis and rabies, and thus contribute to more thorough original investigation of these diseases.

The Army Legislative Committee, through Chairman, J. P. Turner, reported their progress in the last session of Congress and their plans for the coming session. The death of one of their members, Dr. John R. Hart, was most feelingly and fittingly alluded to and how great a loss it was felt to be in this work.

No act of incorporation was yet obtained, but much light was shed for the benefit of the new committee, showing the existence already of a national act of incorporation obtained some years before.

An appropriation of fifteen dollars was made to the Pasteur Monument Fund, and other contributions were solicited.

The presentation of the resolutions prepared for the local committee at Buffalo was reported as completed, and the information afforded that a copy of the same had been placed in the City Hall of Buffalo.

The State Secretaries' reports were read by States and referred to the Publication Committee for abstracting and printing.

The afternoon session of the first day was devoted to the reading of the several papers on "Tuberculosis," the first being that of Dr. T. D. Hinebauch, on "The Action of Tuberculin on Healthy Cattle." A careful *résumé* of a series of experiments accompanied the paper, adding much to its value from a statistical point of view.

Dr. John M. Parker followed as one of the assigned speakers on this subject. His views were prepared in written form, part of which is published in this number, and the completion of which will follow in the succeeding issue. He covered the field of this subject in its several important aspects, and in his conclusions presented a very sanguine belief that its ultimate control and restriction would be accomplished through State aid.

Dr. E. P. Niles then presented the subject from the standpoint of municipal control of tuberculosis, its value, defects, and difficulties. He recommended that all municipalities require an exact supervision of all dairies and the testing of all herds; elimination of all tubercular animals, and central abattoirs for the preparation and sale of all meat.

Dr. Leonard Pearson in his remarks referred to the educational features of the campaign for the extermination of tuberculosis among the live-stock of Pennsylvania. He favored the education of cattle-owners to the dangers of the disease and the advantages to them of having it eradicated. This method of procedure was winning its way in Pennsylvania, and promised excellent results and at a comparatively small expense.

Dr. A. W. Clement referred to the several plans suggested for its suppression through State measures, and believed that much education of the people and those engaged in the live-stock industry was yet to be done. He questioned the right or justice of using State money in paying for diseased and worthless animals.

Dr. D. E. Salmon favored proper supervision of the meat of tubercular animals, and when but slightly affected its use, under proper cooking, was not dangerous, and the saving of this large loss would contribute much to the solution of the control of this all-important problem.

Dr. James Law in his remarks made a strong point for uniform State laws of control and regulation, as the police regulations of one State often resulted in many diseased ones being sent to adjoining States.

Drs. Cary, Stalker, Lowe, Parker, and Gill participated in the discussion, bringing out many salient points.

The deaths of Dr. O. H. Flagg, of New Bedford, Mass., one of the oldest members, and of Dr. John R. Hart, of Philadelphia, Pa., one of the most earnest and regular members in attendance, and Dr. C. F. Douglass, of Jamaica, one of the younger members, were announced, after which adjournment followed until 9 A.M. Wednesday.

The meeting was called to order on the second day at 9 A.M., with President Osgood in the Chair. After receiving the report of the Executive Committee, recommending favorably several applicants for membership, which recommendation was approved, the Chair announced as the Committee on Resolutions, Drs. Pearson, Hinkley, Salmon, Stalker. He also announced the following Committees on Resolutions relative to the deaths of the several members announced on Tuesday: Drs. Pearson and T. B. Rayner, on the death of Dr. J. R. Hart; Drs. R. R. Bell and Ackerman, on Dr. Charles F. Douglass; and Drs. James Law and James B. Rayner, on Dr. O. H. Flagg.

The President then announced that the next order of business would be the election of officers, and appointed as tellers, Drs. H. D. Gill and A. T. Peters. Nominations for President being in order, Dr. C. A. Cary presented the name of Dr. D. E. Salmon, and Dr. William Herbert Lowe named Dr. F. H. Osgood, whose nomination was seconded by Dr. H. D. Gill. Dr. W. L. Williams, in seconding the nomination of Dr. Salmon, advocated the election of the President, for but one term, referring to his own experience as President, of having failed in a reëlection and of receiving much adverse criticism, though he had endeavored to discharge the duties as thoroughly as possible. Dr. Hoskins then took the floor and referred with much pleasure to the names of the candidates and their assurance to fill the chief office well. He desired to state that the failure of the preceding speaker to receive a reëlection was not due to any lack of effort on his part to discharge the duties of President with zeal and ability so far as was within his power; that he had never heard of any adverse criticism of his administration, and that his reëlection failed simply because of peculiar circumstances that had arisen at that time which carried into office one of the officers of the Association who had served for several years in the office of Secretary. In advocating the reëlection of Dr. Osgood he referred to the admirable suggestions made in his opening address, that he felt would contribute very largely to the solution of several serious questions relative to the membership. These plans he would like to see carried into execution. The result of the ballot showed twenty-nine votes for Dr. Salmon and twenty-three for Dr. Osgood, after which the President expressed his good-will in the result of the election by saying: "There is no gentleman in the room whose election I had rather announce then our Chief of the Bureau of Animal Industry." Nominations for the three Vice-Presidents were quickly made: Drs. T. B. Rayner, Eastern Vice-President; W. C. Rayen, Central; and A. T. Peters, Western Vice-President. There being no other candidates named, the nominations prevailed, and the Secretary cast the ballot. The nomination for Treasurer being in order, the name of Dr. James L. Robertson came from a number of sources through the room, but Dr. Hoskins rising to the floor, called the members' attention to the repeated requests from Dr. Robertson in former years to be relieved of the duties of this office, which he had no desire to retain, and that, in view of his absence, it would be unfair to impose these duties upon him again; but if there was any one present who had any knowledge of his willingness to serve again, he would strongly favor his candidacy. On these grounds, the name of Dr. William Herbert Lowe was presented, and also the name of Dr. W. L. Williams by Dr. Tait Butler. Dr. Williams declined the nomination, and the nomination of Dr. Lowe was seconded by Dr. Leonard Pearson, and, there being no other names, the Secretary was instructed to cast the ballot. The nomination for Secretary being in order, by common consent from all sections of the room, the present incumbent, Dr. Stewart, was renominated, and the motion at once prevailed that he cast the ballot for his own reëlection.

The reading of papers then proceeded, as follows: Dr. Leonard Pearson presented the subject of "A Review of the Field of Veterinary Science." This paper exhibited an unusual amount of earnest thought, and was an exhaustive consideration of the future work of the profession, and was listened to with the greatest interest by the audience present.

This was followed by a paper on "Our Milk Supply," by Dr. Charles Ellis, of St. Louis, Mo. This paper was discussed at some length by Drs. O'Connell, Parker, and the essayist.

This paper was followed by Dr. W. H. Dalrymple, on "The Veterinary Field in the South." He referred to the work of education already done by the veterinarians, and of their importance and worth in conserving the health and wealth of the people in the South, and of how much good would be gained by a better knowledge of the treatment, of live stock, internal sanitation, and legislation that would better protect the people of the South. This paper was discussed by Dr. Butler, of Mississippi, who referred to the healthfulness of the climate of the South, the great need of efficient veterinarians, and the character of the Southern people of our country. Colonel Harding, a prominent stock-breeder of Tennessee, spoke of the opening in the South, of the meagre knowledge of the early veterinarians who came to that section, and that there was no section of our country where one would receive a better recognition in proportion to his knowledge and ability than in the South. Drs. Cotton, Heitzman, Jr., O'Connell, Cockerill, Wheeler, and Plunkett, ex-President of the State Board of Health, contributed to the discussion of the veterinary field in the South, all of which shed a light on the social status and the appreciation of the Southerners for talent and education.

Dr. C. A. Cary, of Alabama, then presented the subject of "Osteoporosis," illustrating his paper by the exhibition of many pathological specimens of diseased bones that he had secured bearing on this subject. This everyday topic and condition, so distressing at times to the routine veterinarian, brought a number of the members to the floor regarding their peculiar experiences with epizooty in some sections, the difficulties of diagnosis, interesting methods of treatment and the lack of knowledge of the true cause of the disease. It culminated in this subject being referred to the Committee on Diseases for the ensuing year, with instructions that they obtain data, statistics, and all information possible of this disease, for use in a thorough investigation of this subject. Among those who discussed this paper were Drs. A. H. Baker, Wheeler, Bell, Rectenwald, Hoskins, O'Connell, T. E. White, Gill, and General Cockerill.

*To Belle Meade.* The hour for adjournment having arrived, the Chair announced that the banquet had been fixed for Wednesday evening, and that the members would convene at the railroad depot at three o'clock to accept the courtesy of the railroad company and the Centennial Exposition officers in a trip to the famous Belle Meade Stock Farm. This proved one of the very pleasant features of the meeting. Over one hundred visited this famous farm, and there vied with one another in admiring the beauty and wonderful records of such famous horses as "Iroquois," "Luke Blackburn," "Inspector B," and the grave of "Inquirer," over which Mr. J. R. McLean, of Cincinnati, has erected a beautiful monument to his memory. The excellent and well-kept dairy was also visited, and the method of feeding and the preparation of ensilage was of much interest for all who participated in this outing. General Cockerill contributed much to the pleasure of this trip by the information he furnished of the breeding and records of the horses seen, while Major McCann seemed to be at all points, endeavoring to add to the pleasure of his large number of guests. Before returning to the train almost one hundred assembled on the porch of the famous old homestead, and were there photographed in group form. The ride back to the city was a pleasant one, and many were the kind compliments of appreciation exchanged over this added pleasure of our sojourn in Nashville.

*The Banquet.* At 9.30 o'clock Wednesday evening, around a beautifully decorated table at the Hotel Tulane, there sat down as congenial a body of veterinarians and invited guests to the number of sixty-five as had, perhaps, assembled in Nashville for many years. An excellent *menu* had been provided by the genial proprietor of the hotel, which the guests proceeded to enjoy, and the bounteous repast, from oysters to Maryland soft-shell crab, with Western grouse, frogs' legs, young chicken, and fresh shrimp, mingled with many other delicacies, was improved by a very liberal addition of sauterne, claret, and then capped with champagne. Reaching the latter, the toast-master, Dr. W. Horace Hoskins, rising at his place, introduced to the assembled guests Dr. Price, of the Medical Department of the Vanderbilt University, and asked him to respond to the toast of "How Well Our Sister Profession was Keeping Pace with its Advancing Brother, the Veterinary Profession." Dr. Price proved fully equal to the occasion, and thanking the guests for their very kind invitation, he spoke in very cordial terms and appreciation of the progress of veterinary science and of the close relationship which now existed, making the lines of distinction very narrow indeed between those who devoted their attention to human and comparative medicine. Dr. D. K. Smith was then asked to respond to the toast of "Veterinary Education," and his remarks were listened to with very great pleasure, and many were the warm expressions of delight at the privilege of hearing him and, in the absence of his father, Prof. A. Smith, of Toronto, of having one so well equipped to aid the progress of veterinary education in Canada. "The Responsibilities of a President, and the Many Duties it Carries with it," was most feelingly responded to at the call of the toast-master by President Osgood. "State Veterinary Associations" proved a well-selected topic for presentation by Dr. Nelson P. Hinkley, whose labors and efforts as an ardent association member are well known all over the land. Calling upon the President-elect, Dr. D. E. Salmon, on whose shoulders are to fall the mantle of responsibility for progress from a national point of view during the ensuing year, Dr. Salmon responded, touching upon the work with which he had been identified in other directions and closed with a feeling appreciation of the honor conferred upon him. "How to Obtain State Legislation," was the announced subject for response by Dr. A. H. Baker, who, after referring to the unsuccessful efforts in his own State, most severely criticised legislation that had already been obtained in some of the other States. "Journalism" proved an interesting topic, responded to by Dr. Tait S. Butler, whose experience in the Southern field in journalistic efforts was much appreciated, and his kindly references to the work done in this direction by veterinarians seemed to meet with general approval by all those around the festal board. "State Veterinary Sanitary Work" was the next subject announced, and State Veterinarian Dr. A. W. Clement, of Maryland, was called to respond. He dwelt much upon its great importance and of how much there was to be gained in the better control of all infectious and contagious diseases of animals through properly regulated boards and measures in this direction. The toast-master had reserved for the closing of this very happy occasion one with whom every member felt on the most kindly terms, and vied with one another in gaining his closer friendship, and, calling to the floor Major John J. McCann, for some twenty minutes mirth, gladness, and appreciation prevailed at the continuous flow of wit, satire, and poetry that poured forth with so much grace, ease, and pleasure, and with one accord a unanimous decision followed that the Exposition must have selected for the Chairman of its Entertainment Committee the best equipped Southerner for this place within their borders. A silent toast in memory of the departed members, whose loss of support and assistance the association keenly felt during the past year, was drunk in silence, after which the guests departed to their respective quarters, feeling that it was good to have been there and that this feature of our annual conventions should be strengthened in its influence and its creation of true fraternal feeling.

*Our Last Day.* A long session of the Executive Committee on the morning of the third day resulted in the convention convening at 11 o'clock. The report of the Executive Committee was received and its recommendations approved, after which the subject of "Texas Fever" was presented by Dr. J. M. Connoway, of Missouri. The interesting experiments and the results obtained were fully presented, the importance of this work to the South, to the live-stock industry, and to consumers of the meat-supply afforded by the South were thoroughly presented, and the methods by which it was hoped to control its dangers fully related by Dr. Connoway. The "tick-theory" was presented, and boxes of ticks and many other specimens were passed, after which the methods used to remove and destroy these parasites was referred to, after which the subject was freely discussed in its many important aspects by Drs. Parker, E. P. Niles, Francis, Roberts, Salmon, Law, and Wheeler.

The afternoon session was opened by the presentation of a paper on "Veterinary Education in Medical Colleges," by Dr. E. P. Niles, of Blacksburg, Va. He showed how little attention to the importance of comparative medicine had been exhibited by the colleges in the present century, and how great had been the line of work in this direction in the early history of medicine, and how needful it was if the best results were to be obtained in the sanitary field and in the domain of preventive medicine. The paper was discussed by Drs. Gill and Hinebauch.

"Millet Feeding and the Results Thereof" were presented in a contribution by Dr. Hinebauch, bearing upon the same lines as the subject of his paper at a prior meeting. He presented statistics of considerable importance.

"Inhalation-pneumonia," by Drs. W. L. Williams and P. A. Fish, of Ithaca, N. Y., proved an interesting paper, with some original work in the line of treatemnt, presented with a view of encouraging others to make similar experiments, with the hope that some better results might be obtained in dealing with certain forms and stages of pulmonary diseases. (This paper appears on page 619 of this issue.)

The subject of "Rabies" was then presented by Dr. A. W. Clement, of Maryland, in which he strongly combated the disposition to deny the existence of this disease, after which the paper was discussed by Drs. Salmon, Gill, Cotton, Ellis, Parker, Dalrymple, Hoskins, Plunkett, Meyer, Pearson, and Law. Many diverse views were presented by those participating in the discussion, and the best methods of controlling it and its dangers were fully dilated upon by all the members present.

The several committees on resolutions then presented their reports, as follows:

WHEREAS, it has pleased God Almighty to remove from our midst our fellow-member, Dr. Charles F. Douglass, of West Indies, it is hereby

*Resolved*, that in the death of Dr. Douglass this Association has lost a valued member and true friend of the veterinary profession, and be it also

*Resolved*, that in his death the veterinary profession loses a worker in a country where the veterinary profession can ill afford it;

*And be it further resolved*, that a copy of these resolutions be suitably engrossed, forwarded to his family and published in the veterinary journals of this country.

ROSCOE R. BELL.
E. B. ACKERMAN.

WHEREAS, it has pleased the Almighty to take from us our late fellow-member and associate, Dr. John R. Hart, of Philadelphia; and

WHEREAS, Dr. Hart was intensely interested in the progress of veterinary work and the best development of the profession of which he was an honored member, and his loss has made a most serious void in our ranks, be it

*Resolved*, that we hereby express our deep sorrow and regret and testify to the high character, personal integrity, and generous nature of the deceased, who was unremitting in his efforts to further the aims of this organization, and who cheerfully, with great energy and high ability, performed all duties assigned to him.

*Be it further resolved*, that we hereby tender our sympathy to the bereaved family; and be it further

*Resolved*, that a copy of these resolutions be spread on the minutes of this Association and one sent to the family of our later member.

THOS. B. RAYNER.
LEONARD PEARSON.

WHEREAS, in the providence of God there has been removed from our midst a valued charter member of the United States Veterinary Medical Association in the death of Dr. O. H. Flagg,

*Resolved*, that this meeting hereby expresses its sense of the great loss to the Association and to the community in which he lived, and of which he has long been an esteemed and respected member, and that we tender to his bereaved family the expression of our profound sympathy in their irreparable loss.

*Resolved*, that copies of this resolution be spread on the minutes of the Association and forwarded to his afflicted family. JAMES LAW.

JAMES B. RAYNER.

*Regulation of the Tuberculin-test and Safeguards Needed.*

WHEREAS, the tuberculin-test has been proven to be the only reliable ante-mortem means yet discovered of determining the existence of tuberculosis in its obscure forms; and

WHEREAS, the repetition of this test on the same subject tends to lessen the characteristic reaction, thus producing a non-responsive condition of the animal, which may be mistaken for soundness; and

WHEREAS, these facts may be and are taken advantage of by the irresponsible and unscrupulous to aid in the disposition of diseased animals,

*Be it resolved*, that the private employment of this test except as supervised by competent and responsible persons is fraught with danger to the public.

*Necessity for Better Provisions in the Sale and Exchange of Cattle for Breeding Purposes.*

WHEREAS, the sale of cattle for breeding purposes furnishes abundant and frequent opportunities for the dissemination of tuberculosis to uninfected herds,

*Be it resolved*, that all cattle sold for such purposes should, before going to their destination, have the tuberculin-test applied, and a certificate in accordance with the facts be furnished by the proper sanitary officer of the State.

*Our Reception at Nashville.*

WHEREAS, this the Thirty-fourth Annual Convention of the U.S.V.M.A., now closing its sessions in Nashville, has received every courtesy and assistance from the local Committee of Arrangements, the veterinarians of Tennessee, the citizens of Nashville, the Nashville and Chattanooga Railroad, and the authorities of the Tennessee Centennial Exposition, through their representative, Hon. John J. McCann; and

WHEREAS, this generous and hearty reception has greatly facilitated our work, and has made our visit enjoyable and profitable to an unexceptionable degree, be it

*Resolved*, that the thanks of this Association are hereby tendered the veterinarians of Tennessee and the South and to the citizens of this beautiful city for the hospitality we have enjoyed; to the press of Nashville for the full and accurate accounts of our proceedings, and to Hon. John J. McCann for his untiring efforts that have so largely contributed to our comfort and entertainment.

*To Abolish Small Slaughter-houses and to Establish Central Abattoirs.*

WHEREAS, the small slaughter-houses in the country districts, as well as in cities, are difficult of proper inspection and supervision, as they are so widely separated and are operated irregularly; and

WHEREAS, unscrupulous parties take advantage of this fact to have animals in a diseased and unsound condition slaughtered for food at such places, thereby menacing the public health; therefore

*Be it resolved*, that the U. S. Veterinary Medical Association recommends the prohibition of small and widely scattered abattoirs, and the establishment of central slaughter-houses with municipal ownership, if practicable, where constant and rigid supervision may be enforced.

*Instruction for Committee on Diseases.*

*Be it resolved*, that the Committee on Diseases is hereby instructed to devote special attention to the subject of osteoporosis and rabies; especially to the distribution, prevalence, and losses caused by these diseases, and to report upon the same to the next annual meeting.

*As to the Milk-supply of Cities and Towns.*

WHEREAS, the character of the milk-supply of a city is of the greatest importance to the health of the public, and a contaminated milk-supply is often followed by widespread suffering and numerous fatalities; and

WHEREAS, the wholesomeness of the milk-supply is of equal importance with that of the water-supply, for the improvement of which such intense efforts are frequently made; and

WHEREAS, milk is contaminated in a great variety of ways, some of them so obscure that they are frequently overlooked and require expert knowledge for their detection, and the usual city inspection, consisting merely in the detection of added water, is entirely insufficient for the proper protection of the public, and an inspection is required that includes the observation and correction of defects of all phases and stages of the operation of milk production and distribution, be it

*Resolved*, that a thorough veterinary system of dairy inspection is hereby recommended to all Boards of Health, with the belief that it will exert an important influence for the protection of public health, the preservation of infant life, and the betterment of the diet of the people through the increased consumption of safe and wholesome dairy products.

*As to Rabies.*

WHEREAS, rabies of dogs and other domestic animals prevails in certain sections of the United States; therefore

*Resolved*, that the sanitary authorities of the various localities should take some action looking to the control of dogs for the prevention of the disease.

*The Merit System of Appointment.*

WHEREAS, the growth and expansion of the civil service, or the merit system of appointment, has received our official approval from time to time; and

WHEREAS, its continuance has demonstrated the wisdom and value of this method of selection for the public service, and has secured the best corps of workers the public has ever had in its employ, be it

*Resolved*, that we most earnestly urge its continuance, its higher development and further extension, and that we pledge our united support to our National, State, and municipal officials who are charged with its execution and maintenance.

Papers on "Bacteriology," "Malignant Catarrh of the Ox," "Use of the Actual Cautery," "Radical Operation for Contracted Hoof," "Infectious Mammitis," "Some Diseases of the Horse Peculiar to Mountain Regions," were read by title and ordered placed in the hands of the Publication Committee.

The following places were then advocated for the meeting of '98: Boston, Omaha, St. Louis, and New York City. The outgoing President made a short speech, thanking the members and others for their attention and assistance to the Chair throughout the convention, and introduced in very kind words his successor, Dr. D. E. Salmon, who, in taking the Chair, feelingly acknowledged his appreciation of the very high honor conferred upon him, and asked for the same kindly support from the members that had been accorded his predecessor. The newly elected Vice-Presidents, Secretary, and Treasurer were introduced, after which the now famous meeting of '97 closed.

### ASSOCIATION OF VETERINARY FACULTIES.

THE meeting was held on September 8, 1897, at 8 P.M., in Tulane Hotel, Nashville, Tenn., during the meeting period of the United States Veterinary Medical Association, Dr. Pearson in the chair. The following colleges were represented: Harvard University, Dr. F. H. Osgood; American Veterinary College, Dr. R. R. Bell; New York College of Veterinary Surgeons, Dr. H. D. Gill; University of Pennsylvania, Dr. Leonard Pearson; Columbian University, Dr. D. E. Salmon; Kansas City Veterinary College, Dr. S. Stewart; McKillip Veterinary College, Dr. F. S. Schoenleber; New York State Veterinary College, Dr. James Law; Iowa Agricultural College, Dr. M. Stalker; United States Veterinary College, Dr. C. B. Robinson; Ontario Veterinary College, Dr. D. K. Smith; Chicago Veterinary College, Dr. A. H. Baker.

Drs. Hoskins, McKillip, Clement, and Parker were also present, the last two being invited to be present as representing McGill University unofficially. On motion of Dr. Stewart, seconded by Dr. Baker, the minutes of the last meeting were approved as read.

The Executive Committee reported favorably on the application of Dr. R. R. Bell, American Veterinary College; Dr. Greiner, Indianapolis Veterinary College; Dr. Smith, Jr., Ontario Veterinary College; and Dr. F. S. Schoenleber, McKillip Veterinary College. Drs. Clement and Parker, not being eligible to represent McGill University (not of the Faculty or teaching staff), were reported unfavorably. On motion of Dr. Baker, seconded by Dr. Stewart, that those representing other schools, but not eligible to vote, be invited to attend the meeting during the reading of papers and the discussion of the same; carried.

Dr. Salmon, of the Committee on Uniform Standard of Entrance Examination (Drs. D. E. Salmon and James Law), stated that he had no report on the subject, but that Dr. Law had. Dr. Law read his report, and it was discussed at length by every member present, and the discussion waxed so warm that Dr. Law protested to the very pointed and personal remarks of Dr. Robinson, who was repeatedly called to order. When asked if the report of Dr. Law expressed the ideas of the whole committee, Dr. Salmon answered the chair that it did not, and that he would present one at the next meeting. On motion Dr. Law's report was accepted, after it had been explained that acceptance does not imply an indorsement of the views expressed. On motion, further discussion was postponed till after the reading of Dr. Hoskins' paper on "Uniformity of State Regulations Governing the Practice of Veterinary Medicine." Both papers were freely discussed. The prevailing opinion seemed to be for uniform entrance examination. On motion Dr. Hoskins' paper was accepted. On motion of Dr. Osgood, seconded by Dr. Schoenleber, that a committee of five, as follows, Drs. Osgood, Law, Salmon, Schoenleber, and Bell, be appointed to report at the next meeting on the advisability of forming a "National Examining Board," was amended by Dr. Salmon, seconded by Dr. Bell, that a provision should be made for a coöperation with State associations to secure a uniform provision in legislation authorizing the examining board to establish the standard of examination, and to accept the certificate of other boards that have satisfactory requirements. Motion as amended was carried.

The following amendment to Articles I. and II. of the Constitution was proposed by Dr. Pearson: *Resolved*, That Article I be so amended as to read that this body shall be known as "The Association of Veterinary Faculties and Examining Boards of North America;" that Article II. be so amended as to read, "This Association shall consist of members of governing faculties and teaching-staffs of all veterinary schools of North America which confer a degree in veterinary science, and of members of State veterinary examining boards that grant licenses to practice; but each college and board shall have but one vote in the meeting, and that vote shall be cast by the properly appointed delegate of the school or board." The President ordered it to take the regular course.

The election of officers being in order, on motion of Dr. Robinson, seconded by Dr. Osgood, the present incumbents were declared re-elected, and the Secretary cast the ballot as follows: Dr. Pearson, President; Dr. Gill, Secretary-treasurer; Drs. Law, Stalker, McEachran, Salmon, Robertson, Executive Committee.

On motion of Dr. Osgood, seconded by Dr. Robinson, that minutes of the meeting and papers be published in the report of the United States Veterinary Medical Association; carried.

Meeting adjourned. H. D. Gill,
Secretary-Treasurer.

### PENNSYLVANIA STATE VETERINARY MEDICAL ASSOCIATION.

The semi-annual session was convened in Franklin, Venango County, on Tuesday, September 21, 1897. The commodious and attractive apartments situated over 1249-1251 Liberty Street, and known as the A. O. U. W. Hall, had been selected by the local reception committee. Here was held one of the most interesting and successful meetings experienced by the Association in the history of its autumnal sessions. An incident occurred in the early morning that proved a delightful experience to the attending members. The Association received an invitation to visit the Prospect Hill Stock Farm of Messrs. Miller & Sibley. Mr. E. H. Sibley gave inspiration to his invitation by sending two elegant four-in-hands to convey the guests from the Exchange Hotel to the farm. To those who were strangers in this unique and prosperous niche in the great oil regions a tally-ho drive through a section whose hillsides are dotted with scores of derricks over oil wells was a novel experience. The enjoyment to all was as keen as the morning was beautiful and the scenery picturesque and inspiring. The inspection of the trotting stock and stud, also the magnificent herd of high-class Jersey cattle, greatly interested the veterinarians, who are lovers of quality in stock and who appreciate the beauty and value of hygienic surroundings. Much praise and satisfaction were expressed with what was seen, and many thanks were tendered Mr. Sibley for his attentive courtesy extended the visiting members through Mr. Gray, his representative.

Returning from the farm, the Association was called to order at 11.30 A.M. by the President, James B. Rayner. On motion, the roll-call was dispensed with, and the Recording Secretary at once proceeded to read the minutes of the previous meeting, which were approved as corrected.

The President then delivered his address. He thanked the Association for the honor conferred by electing him as its presiding officer for the ensuing year. He spoke with enthusiasm upon the value to the profession of veterinary associations. He showed them to be powerful educators and the means of stimulating the best elements in and elevating the character of the profession. He spoke of the value of fraternal relationship, the additional strength given the State and national associations by the local ones. He said that a closer affiliation of associations is necessary to give the best support to the important questions upon which we deliberate and aim to influence legislation. Advancement is the result of personal interest, no less in the history of veterinary professional work than along other lines, and that if each member would contribute papers, reports, and personally work for the interest of the Association, the latter is sure to be successful. The President pointed with pride to the position of the veterinary student of to-day contrasted with said position thirty years ago. He had witnessed with delight the astonishing progress in the growth of the profession and in the opportunities afforded by colleges for securing a veterinary education. He said: "You are so much better prepared, so much better provided for than we who have preceded you, that we can pass from the duties that have been ours with strong faith that you will carry on the good work. As one with long experience, I can say that though the duties are hard at times, they are pleasant. They are worthy of your best efforts. With an honest purpose to do right, and earnest labor for those who place their confidence in you, you will be successful and will shed honor on your chosen profession."

At the close of his address the President introduced the Mayor of Franklin, George B. Jobson, V.S., who, in a happy strain, spoke cordial words of welcome. The Mayor dwelt upon the progress and status of our profession; of its age in the world; its prestige; the immense value the study of comparative medicine had been to the progress of human medicine on essential problems; its conspicuous place as the guardian of the health of the people in being able to provide a healthful meat- and milk-supply as well as to protect the community from the ravages of certain contagious diseases. He complimented the Association on its good work; on the creation of the State Veterinary Examiners' Board to influence higher education and take the profession out of the hands of empirics; on the State Live-stock Sanitary Board to promote the health of the people of this great Commonwealth and to conserve the moneyed interest in live-stock in our State. The Mayor by his hearty greetings made each member feel that he was at home in Franklin.

Following the Mayor's address, Dr. W. Horace Hoskins made fitting remarks upon the character and usefulness of our late member, Dr. John R. Hart, and recommended that a committee be appointed to draft resolutions of respect and condolence. The chairman then appointed Drs. Hoskins, Pearson, and T. B. Rayner to constitute said committee.

Election of officers being next in order, Dr. Hoskins presented the name of Dr. Francis Bridge for Treasurer, to fill the vacancy caused by the death of Dr. Hart. On motion Dr. Bridge was unanimously elected.

The following names were proposed for membership in the Association: Henry Bowers, V.M.D., Philadelphia; Samuel J. Swift, D.V.S., Franklin, Pa.; W. E. McCray, V.S., Oil City; Harry Emery, D.V.S., Pittsburg; David Waugh, D.V.S., Pittsburg; E. C. Porter, V.S., Newcastle; E. E. Bittles, V.S., Newcastle; M. J. Chrisman, V.S., Sugar Grove; A. W. Weir, V.S., Greenville.

To fill vacancies on the Board of Trustees the chairman appointed Drs. Hoffman, Michener, Rectenwald, and James A. Waugh. Following a short intermission the chairman of said board reported favorably upon all the applications for membership. The board in its report considered the relation to this Association of members who move to other States to practise their profession. While the board holds that such members may retain their membership in this Association, if they so desire, it urgently recommends them to ally themselves with the interests of the Association of the State in which they practise. The above recommendation was adopted.

The following resolutions were prepared and adopted by the Association:

*Whereas*, Of the last three meetings of the U. S. V. M. A. two have been held in the West—*i. e.*, in Des Moines and Nashville—and the third at Buffalo, three hundred and fifty miles from the seaboard; and

*Whereas*, The veterinary profession in the East has been somewhat neglected, and has suffered from such oversight, and needs the stimulation and cohesive influence of a meeting of the National Association; be it

*Resolved*, That the Pennsylvania State Veterinary Medical Association earnestly petitions the President and officers and other members of the Executive Committee of the U. S. V. M. A. to favor the holding of the next meeting at Boston, and expresses the belief that it would be a serious error to hold three or four meetings a long distance from the centre of membership of the Association, and especially to select a meeting-place in a territory that has been occupied within three years.

Following the discussion and adoption of this report, the Board of Trustees having nothing further, on motion, the Association instructed the Secretary to cast a ballot for the new members, who were then introduced by Dr. Jobson.

*Corresponding Secretary's Report.*—In his written report, after showing his expense account, the Secretary dwelt upon his correspondence, not only with each member of the Association, but with every honorable veterinarian in this State and the adjoining counties in New York and Ohio. The number of replies and inquiries from them serves to indicate the interest awakened by association work among new members and its wholesome influence upon the great body of professional men in veterinary medicine. A number of complaints of illegal practice carried on in numerous ways and by various ruses have been reported. These have been turned over to the State Board of Examiners, under whose jurisdiction such matters come.

On motion, made by Dr. Rhoads and carried, a committee of two was appointed to examine and assist in settling the Association accounts of the late Treasurer before said accounts pass into the hands of the newly-elected officer. The President appointed Drs. Hoskins and Pearson to constitute said committee.

Under the head of new business, Dr. Noack asked why the State Association of Pennsylvania and the National Association convened in the same month. He pointed out the inconvenience, the impossibility in some cases, of attending both meetings, and suggested that a difference of several months be made between them. This opened a lively discussion. It was shown that the time of the semi-annual meeting of the State Association had been changed from the Tuesday after the first Monday in September to the third Tuesday in the month, to overcome this embarrassment; that the date of the National Association was one most favorable to its members, many of whom could not otherwise attend; that September was a favorable and pleasant month to hold conventions; that we have no jurisdiction over the National Association, and that an idea contemplating the radical change of the time of the meetings of this Association would be more practical before the annual spring session at Philadelphia.

*Report of the Committee on Legislation.*—Dr. Leonard Pearson, as chairman of the Committee on Legislation, in a verbal report, in effect said: "One difficulty encountered by the State Live-stock Sanitary Board in its efforts to deal with the suppression and prevention of tuberculosis in the past year was how to prevent the disease from being reintroduced on premises by cattle brought from other States or counties for dairy or breeding purposes. In other words, how were we to protect our cattle interests against a supply being brought from an infected source? It had been suggested that legislation be secured to control the admission of cattle into the State, admitting only such as could be shown to be free from tuberculosis. Such a law had since been enacted. It provides that cattle coming into the State to be used for dairy and breeding purposes must undergo the tuberculin-test before admission. The test may be made at the most convenient stock-yards on the line of railroad over which the cattle are shipped or in quarantine. The law takes effect January 1, 1898. Said law is enforced by the Live-stock Sanitary Board, and a fine of fifty dollars is imposed for its infringement. In flagrant cases of its violation the punishment may extend to imprisonment; but the details of the method of enforcing the law and of inspection and reporting violation, etc., had not been fully developed, but would be brought to completion in the near future. Dr. Pearson declared that this law was a necessity. It was not a new idea, for other States had adopted similar measures and required the tuberculin-test. Of the New England States this is especially true, and Pennsylvania would not be made a dumping-ground for diseased animals that could not be disposed of in other Commonwealths.

Another measure has been passed appropriating $15,000 to be expended in the investigation of infectious and contagious diseases. The value of the live-stock interest of Pennsylvania aggregates approximately $125,000,000. The loss sustained through contagious diseases is enormous, and is expressed in millions. The object of this measure is to lessen these great losses by prevention of the plagues that produce them; but the particular purpose is to prevent tuberculosis. It would seem a prodigious task to apply the tuberculin-test to all the cattle in this State. It would be impossible from the standpoint of cost. To prevent the malady would appear to be the most practical method. But how? We believe that in a more perfect knowledge will be found the means we desire. We propose to study these great problems experimentally, and to determine under what causes and conditions infectious and contagious diseases are spread. Thus we hope to arrive at more positive knowledge, more definite conclusions than we now possess. We will study the progress of tuberculosis under both sanitary and unsanitary conditions. The elements of light, darkness, moisture, and temperature will be observed in their effects upon animals in health and disease and their relation to the propagation and spread of the same. With proper facilities we may study the disease under a variety of conditions at one and at the same time. The amount set apart for such study is scarcely adequate, but it marks a beginning. This movement we now make is an innovation. In Nebraska there is a similar one, but this is among the first of its kind in this country. Views concerning the phenomena of causes, progress, and spread of contagious and infectious diseases are numerous; but they all lack accurate, scientific proof; hence the necessity for such data. Other diseases may be studied at the same time, viz., osteoporosis, about which there are so many theories, yet little positive knowledge; also the so-called cerebro-spinal meningitis, contagious abortion, anthrax, and hog-cholera. It is largely due to your influence—to the influence of this Association—that the step is taken. It is not more than fair that you should have a voice in this matter and suggest the means that may solve this problem before us. I invite discussion, and hope you will be free to make suggestions, and thus help forward the work you have begun.

At this time a motion to adjourn was made and carried. A bounteous repast had been prepared in the room adjoining the hall. The veterinarian does not take a back seat, if judged by the old saying, "A man who cannot eat cannot work."

The afternoon session was called to order at 2.20 oclock. The roll-call showed the following members present: Drs. Hoskins, Hoffman, Helmer, Irons, George B. Jobson, George Jobson, McNeil, Michener, Noack, Pearson, Thomas B. Rayner, James B. Rayner, Rectenwald, Rhoads, Waugh, and McLean. New members present were Drs. Bittles, Christman, Emery, McCray, Swift, and Wier. The visitors were W. A. Meredith, V.S., and James McMahan. The Corresponding Secretary read letters from several absent members.

*Reports of County Secretaries.*—Representing Venango County, Dr. George Jobson rendered the following report: With the exception of a number of cases of strangles and influenza of a mild type, no diseases of a contagious nature have been reported in Venango County during the past six months.

Montgomery County, by Dr. N. E. Reinhart, of Pottstown, Pa.: His report shows one case of glanders. The animal was destroyed and the premises thoroughly disinfected. The trough, rack, stall, blankets, buckets, in fact, everything used about the animal, were burned and the walls twice white-washed. The doctor has inspected ten herds of cattle since June, and found tuberculosis in each herd. From one to four head in each herd inspected were condemned and destroyed. Dr. Reinhart holds that there are other herds diseased, of which we are ignorant because the owners do not report them. Until we are empowered by law to examine for ourselves, and until cattle are tested with tuberculin when brought into the State, we may expect to have tuberculosis with us. The doctor also reports two cases of spinal meningitis in one stable. Both died within one week from the time of the attack. Other horses were removed to a shed a short distance from the barn. There were no more cases.

*Report of the Commiteee on Sanitary Science and Police.*—Dr. J. Curtis Michener (chairman) made the following report: There are several plans of control work concerning tuberculosis that have been tried in several States:

1. Inspection of all cattle in the State, as in Massachusetts. This is being abandoned on account of its unpopularity with the farmers and dairymen.

2. Inspection here and there as the disease is reported to exist. This is without method and of very limited good.

3. Under direction of State Boards of Health, as in New York State. It is a health measure concerning people and unpopular with farmers.

4. Under direction of State Live-stock Sanitary Boards, as in Pennsylvania.

The State Live-stock Sanitary Board of Pennsylvania consists of four members—Governor, Secretary of Agriculture, Dairy and Food Commissioner, and State Veterinarian—all State employés, but receive no extra pay for serving on the Sanitary Board. The method of work is as follows:

1. Application for inspection, which is a contract, binding the cattle-owner to coöperate in getting rid of the disease and the disinfection of premises.

2. Inspection done at the expense of the State.

3. Cattle appraised and killed.

4. Premises disinfected.

All those whose property is becoming consumed by tuberculosis can avail themselves of these provisions, and get rid of the disease and receive fair remuneration for cattle found diseased and destroyed; therefore, the work receives the active coöperation of the farmers, and results in much permanent good to the State. Disease is being suppressed—in some places rapidly, in others slowly, and without interference with the live-stock and dairy interests. Increased confidence is given consumers of dairy products as well as meats. The public is being educated in the means to be used to prevent the spread of the disease.

Up to the end of the last fiscal year, June 1, 1897, the expenditures for cattle amounted to $43,476.50. Amount paid for inspections, $8291.99. Inspections cost less than 17 per cent. Cattle-owners receive 83 cents on every dollar expended for tests and for cattle killed.

More than 10,000 head have been tested with tuberculin. Less than 20 per cent. of these were found tuberculous. These are herds that were thought infected before being tested. Many of the principal breeding herds have been put on a sanitary basis.

Everything seems to be working satisfactorily, and there seems to be no reason for a change of method. The work is enforced with judgment and conservatism, and promises to free the herds from this destructive disease without creating alarm. Portions of the State that less than one year ago were in bitter hostility, and petitioned the Sanitary Board to stop what they called the wholesale slaughter of herds on the strength of the tuberculin-test, are now perfectly reconciled and are coöperating with the authorities in a good and sure work.

Regard for propriety would require that our report end here, as our knowledge of the occurrences and discoveries in the field of sanitary science and police since our last meeting is limited to these facts, for which we are indebted to our State Veterinarian; but, with your indulgence, we will give ourselves free scope, and glance over the past, present, prospective future. In looking back to student-days of thirty-three years ago, it seems almost incredible that there should be such a wide difference in the actual and sanitary knowledge of the medical world, especially in the veterinary practice of this country. True, in those days Massachusetts had already adopted measures for stamping out contagious pleuro-pneumonia among cattle, and was followed up by other States, and finally brought to a successful termination by the coöperation of our National Government. Glanders was combated and somewhat restrained by a common instinct for the public safety by common law and the aid of a few qualified practitioners. The germ-theory of disease was being accepted by the medical mind, but it has been within a few years that the successful laboratory work has been done and the course determined by which we shall conquer disease and stay pestilence. The progress made in twenty-five years is, indeed, most gratifying and encouraging. It is our privilege, at this time, to point out ways in which we, as practitioners, may aid the good work. Our State, with many others and the National Government, is now in a position to guard our immense live-stock interests and promote sanitary science. Among the chief aids required at this time is more money at the disposal of our Bureau of Animal Industry, the State Live-stock Sanitary Board, and boards of health. We trust that returning prosperity, a better public conception of the needs, and a better realization of the truthfulness of the old saying, "an ounce of prevention is better than a pound of cure," will meet this paramount requirement when the funds are wisely and economically expended. The next important need is more interest and better coöperation on the part of practitioners. They form the chief and most reliable source of information to the State veterinarian and Live-stock Sanitary Board, and are its trusted agents in carrying on the work. We should be well informed. I find it to be an actual fact that we have practitioners who do not know of our State provisions for suppressing outbreaks of contagious diseases and the investigation of causes. We should be in close touch and sympathy with the authorities in their efforts to protect the stockman's interests and the public safety. Our veterinary colleges are furnishing a constantly increasing need in the form of qualified veterinarians to act upon boards of health and as inspectors of live stock and their products, and as public educators and authorities upon which the people can rely. A great work is yet before us until we have the State

Live-stock Sanitary Board so well aided throughout the entire State as to enable it to completely fulfil its mission. In our opinion, it could profitably take cognizance of the most common contagious and infectious diseases, such as strangles and influenza in horses. These diseases inflict an unnecessary amount of loss and inconvenience, due to the ignorant and careless manner in which diseased horses are allowed to mingle with sound ones, and the neglect of disinfection. These diseases are frequent and widely distributed in our part of the country. For instance, a drove of horses is shipped from one distant point to be sold at public sale. The cars they are brought upon and the yards and stables they pass through are ofttimes hot-beds of infection, and when sold they are ready to develop the disease. They are spread broadcast, and each stable in which they go becomes another centre of infection. It need not nor should not be, if proper supervision and restrictions were placed upon the horse-market. The world is starting upon an era of warfare upon disease-germs in animal and vegetable life, and upon injurious insects and noxious weeds, that will some day make this a better sphere to live upon. In this grand battle the veterinarian will stand among the foremost.

The literary programme was now opened with a talk by Dr. George B. Jobson on "Bacteria." Dr. J. B. Irons presented the subject of "Anthrax." Dr. Willis B. McCray, "Parturient Apoplexy;" Dr. W. Horace Hoskins, "Why We Come to Franklin;" Dr. C. C. McLean, "Dairy and Milk Inspection;" Dr. Otto G. Noack, "Report of Surgical Cases, as Tenotomy and Resection of Necrotic Parts of Soft Tissue of Frog Caused by Nail Wounds;" Dr. E. Mayhew Michener, "Record of a Few Cases of Abdominal Surgery." These interesting and valuable papers will all be published in the JOURNAL as soon as room can be found for them. The paper by Dr. C. C. McLean on "Dairy and Milk Inspection" was an exhaustive treatment of the subject. The doctor's experiments before the Association, showing how to detect foreign substances and adulterations in milk, were very interesting and instructive. Dr. Hoskins proposed that this paper be given to the JOURNAL, and that three hundred copies be printed to be distributed among the members of the Association. On motion of Dr. Pearson, Dr. Hoskins' proposal was accepted. On motion of Dr. Waugh, the paper is also to be published in the *Review.*

*Reports of Cases.*—Dr. J. C. Michener read a report entitled "Four Interesting Cases at Cloverdale Farm." This will be published. Dr. McLean reported a rare case. He was called to attend a collie bitch that gave birth to nineteen puppies. The family seemed healthy. Dr. Rectenwald and others reported cases.

The following resolution was presented and unanimously adopted:

*Whereas,* this meeting of the State Veterinary Medical Association has been one of the most enjoyable and successful that has ever been held by this organization; and

*Whereas,* this gratifying condition is due to the energy and courtesy of our fellow-members, the Mayor of Franklin and his associates on the Committee of Arrangements, to the entertainment and instruction afforded by Messrs. Miller & Sibley, to the efforts of those who prepared papers for this occasion, and to the local and county press; be it

*Resolved,* that the thanks of this Association are hereby extended to all of those who have contributed so materially to the success of this meeting, and we hereby express our deep appreciation of the same.

This resolution was also offered:

*Whereas*, we have learned through the State Veterinarian of certain contemplated plans for the better solution of many of the very important problems yet to be solved in relation to the propagation, means of extension, and methods of control of certain infectious and contagious diseases of live stock, especially tuberculosis; and

*Whereas*, these are of the greatest importance to the welfare and health of the public and to the vested interests of a large portion of our people; therefore, be it

*Resolved*, that we most heartily indorse these proposed plans of investigation, and earnestly recommend their early application, that the results to be obtained may be promptly realized.

W. HORACE HOSKINS,
J. CURTIS MICHENER.

Following these resolutions Dr. Hoskins read the by-laws as revised. The articles were read and voted upon separately, and then the whole was adopted as revised.

By consent of the Association a paper was read by Dr. W. A. Meredith, of Corry, Pa., on "Cerebro-spinal Meningitis." Owing to the lateness of the hour (7 P.M.) this paper was not discussed, but a motion to adjourn was made and carried.

Most of the members were obliged to remain in Franklin over night and take early morning trains to their respective homes. Each took with him pleasant memories of Franklin, of fraternal greetings and pleasure in another experience of the days of "Auld Lang Syne."

JACOB HELMER, D.V.S.,
Secretary.

### ECHOES OF THE MEETING AT FRANKLIN.

The Keystone State Association eclipsed all records of former semi-annual meetings in adding to its membership and the scope of the programme at Franklin.

It was an outpouring of the profession from the northwestern tier of counties of Pennsylvania, and more than verified the most sanguine hopes of those who advocated Franklin for our semi-annual gathering.

President Rayner found it necessary to keep things moving along at a fast pace in order to get through with an overloaded programme.

Mayor Jobson exhibited excellent qualifications as a presiding officer, and seems to fill well every duty placed upon him, whether those of a veterinarian, mayor, or as presiding officer of an assembly.

It was Dr. J. Curtis Michener's first visit to Western Pennsylvania, and every hour of the trip was one of enjoyment.

President Rayner's looks like a record-breaking administration for new members.

Secretary Rhoads lost no opportunity of impressing on all visiting veterinarians the value of association privileges, and won many new members.

Philadelphians found the trip *via* Pittsburg and returning *via* Corry the longest railroad ride in the State, but they all returned home feeling well repaid in the good work accomplished.

Recording Secretary Helmer wonders if such an avalanche of work for his office is to continue. It was rather a serious matter in a new office to keep pace with the proceedings.

